# Anti-GAD antibody biochemical correlates of autism symptoms in children: a case-control study

**DOI:** 10.3389/fpsyt.2026.1727227

**Published:** 2026-03-30

**Authors:** Burak Kamış, Perihan Çam Ray, Gonca Gül Çelik, Hülya Binokay

**Affiliations:** 1 Department of Child and Adolescent Psychiatry, Adana City Training and Research Hospital, University of Health Sciences, Adana, Türkiye; 2 Department of Child and Adolescent Psychiatry, Faculty of Medicine, Çukurova University, Adana, Türkiye; 3 Department of Biostatistics, Faculty of Medicine, Çukurova University, Adana, Türkiye

**Keywords:** anti-GAD antibodies, autism spectrum disorder, autistic regression, case-control study, iron deficiency, immune dysfunction

## Abstract

**Purpose:**

To evaluate whether anti–glutamic acid decarboxylase (Anti-GAD) antibodies and selected biochemical parameters are associated with autism spectrum disorder (ASD), symptom severity, and autistic regression in a case–control design. We hypothesized that Anti-GAD titers would be higher in ASD and associated with greater symptom burden.

**Materials and Methods:**

Children aged 2–9 years with ASD diagnosed according to DSM-5 criteria and age- and sex-matched healthy controls were included. Laboratory analyses assessed Anti-GAD, ASO, ferritin, iron, vitamin D, vitamin B12, thyroid-stimulating hormone, and folate. Clinical evaluations included the Autism Behavior Checklist (ABC), Modified Checklist for Autism in Toddlers (M-CHAT), and Ankara Developmental Screening Inventory (AGTE). Autistic regression was defined as loss of previously acquired language and/or social communication skills lasting ≥3 months between 15 and 30 months of age.

**Results:**

Ninety children participated (45 with ASD and 45 controls). Anti-GAD antibody levels were higher in the ASD group than in controls (p = 0.003). Although statistically significant, the absolute difference between groups was modest. Among ASD cases, 62% met criteria for regression; this relatively high proportion may reflect the clinic-based sampling and the operational definition applied. Compared with those without regression, children with regression had higher Anti-GAD titers and ABC scores but lower iron and ferritin levels (all p < 0.05). Anti-GAD levels correlated positively with ABC total scores (r = 0.724, p = 0.01). These findings reflect statistical associations and do not imply causality.

**Conclusion:**

Higher Anti-GAD antibody levels were statistically associated with ASD, autistic regression, and greater behavioral symptom severity in this sample. These results suggest possible immune-related contributions to ASD heterogeneity; however, larger longitudinal studies are needed before clinical or biomarker implications can be established.

## Key Points

Anti-GAD antibodies and biochemical parameters may be associated with autism spectrum disorder (ASD) symptoms.
Children with autistic regression demonstrated higher Anti-GAD titers and more severe behavioral symptoms, but lower iron and ferritin levels compared with those without regression.
Immune-metabolic alterations may contribute to the heterogeneity of ASD.
Anti-GAD antibodies may be associated with biologically distinct ASD subgroups, although their clinical biomarker utility remains uncertain.
Further studies with larger, longitudinal samples are needed to clarify the clinical relevance of these findings.

## Introduction

Autism Spectrum Disorder (ASD) is a neurodevelopmental condition characterized by impairments in social communication, as well as restricted and repetitive behaviors that typically manifest in early childhood (1). First described by Leo Kanner in 1943 (2), ASD currently has a prevalence of approximately 1 in 44 children, according to the 2021 Centers for Disease Control and Prevention (CDC) report (3). ASD is four to five times more frequent in boys than girls, and girls are often diagnosed later and present with more severe behavioral problems and intellectual disability (4, 5).

Genetic, epigenetic, and environmental factors contribute to the etiology of ASD. Twin studies suggest heritability is about 80%, with a concordance rate of around 40% in monozygotic twins (6). However, genetic predisposition alone does not fully explain the marked clinical heterogeneity of the disorder. Increasing evidence suggests that environmental influences and immune–metabolic factors may interact with genetic vulnerability, potentially affecting neurodevelopment through altered neurotransmission, oxidative stress, inflammatory pathways, and endocrine regulation (7–9).

Several prenatal and perinatal factors have been associated with increased ASD risk, including advanced parental age and pregnancy-related complications (10–13). However, these associations do not fully account for the biological variability observed among individuals with ASD. Therefore, research has increasingly focused on potentially modifiable biological mechanisms, including immune dysregulation and metabolic alterations, that may contribute to symptom expression and clinical heterogeneity.

## ASD and regression

Regression in ASD has been described in various ways, but no universally accepted definition currently exists. The most common description involves a loss of previously acquired language and social communication skills, usually during the second year of life (14, 15). Regression is most frequently reported between 15 and 30 months of age (16). A recent meta-analysis estimated the prevalence of regression in ASD to be approximately 32% (16). Although regression is not considered a distinct diagnostic subtype, it represents a clinically meaningful manifestation of ASD heterogeneity. Its occurrence may reflect differences in underlying biological pathways, including genetic vulnerability, comorbid medical conditions, or potentially immune-related processes (17).

## Autism and the cerebellum

The cerebellum plays a critical role in motor coordination, as well as in cognitive and affective processing through its extensive connections with cortical and subcortical networks (18–21). Alterations in cerebellar structure and connectivity have been reported in individuals with ASD, suggesting possible involvement of cerebellar circuits in symptom expression rather than indicating a single causative mechanism (18).

In ASD cases, selective loss of Purkinje cells and atrophy of cerebellar lobules have been observed (22–25). These findings are commonly interpreted within the framework of disrupted excitatory–inhibitory balance.

Given the high density of GABAergic neurons in the cerebellum, alterations in inhibitory signaling pathways have been proposed as one potential mechanism underlying these observations (26, 27). However, the precise contribution of cerebellar changes to ASD pathophysiology remains incompletely understood.

## Glutamic acid decarboxylase

Glutamic acid decarboxylase (GAD) is an enzyme that catalyzes the conversion of glutamate to γ-aminobutyric acid (GABA) and carbon dioxide 9 (28). The GAD enzyme is divided into two isoforms, GAD65 and GAD67, based on their molecular weights, intracellular localization, and enzymatic activities (29). GAD65 primarily regulates synaptic neurotransmission by producing GABA, whereas GAD67 maintains intracellular GABA levels and is essential for synaptogenesis and neuronal protection (30, 31).

The most commonly used autoantibodies in clinical practice are directed against GAD65 (32). Anti-GAD antibodies have been associated with several autoimmune and neurological disorders, including type 1 diabetes mellitus, cerebellar ataxia, limbic encephalitis, and temporal lobe epilepsy (33). Their heterogeneity is explained by different epitopes within the catalytic domains of the GAD enzyme (34).

GABAergic neurons are densely found in the cerebellum, basal ganglia, hippocampus, brainstem nuclei, and spinal gray matter. They are part of an extensive inhibitory interneuron network in the nervous system (35). Experimental studies have demonstrated that anti-GAD antibodies may interfere with presynaptic GABA synthesis or release, potentially reducing inhibitory signaling (36, 37).

In ASD, reductions in cerebellar Purkinje cells and decreased GAD activity have been consistently reported (38, 39). It has been proposed that immune-mediated mechanisms affecting GAD activity could influence GABAergic balance. However, direct evidence demonstrating that circulating Anti-GAD antibodies cross the blood–brain barrier or causally alter GABA levels in ASD is currently limited.

Alterations in excitatory–inhibitory balance have been hypothesized as one contributing factor in ASD-related neurobiology (40). Animal models with reduced GAD67 expression exhibit behavioral features resembling ASD (41); however, translational implications for circulating Anti-GAD antibodies in human ASD remain uncertain.

## Aims and hypotheses

This study aimed to investigate the relationship between clinical autism spectrum disorder (ASD) features and laboratory data, including Anti-Glutamic Acid Decarboxylase (GAD) antibodies, Anti-Streptolysin O (ASO) levels, and biochemical parameters (ferritin, iron, vitamin D, vitamin B12, TSH) in individuals diagnosed with ASD, compared to healthy children, as well as between ASD cases with and without regression.

In particular, we examined whether Anti-GAD titers differed between groups and whether Anti-GAD levels were associated with behavioral symptom severity and the presence of autistic regression within the ASD cohort.

We hypothesized that Anti-GAD titers would be higher in children with ASD than in controls and that higher Anti-GAD levels would be associated with greater behavioral symptom severity and autistic regression.

This study was designed to explore clinically measurable immunological correlates of ASD rather than to establish a causal mechanism.

## Materials and methods

### Participants

Children aged 2–9 years who met the Diagnostic and Statistical Manual of Mental Disorders, Fifth Edition (DSM-5) criteria for Autism Spectrum Disorder (ASD) were included in the study. Healthy children served as the control group.

Participants with ASD were consecutively recruited from the outpatient Child and Adolescent Psychiatry Clinic of Çukurova University Faculty of Medicine between April 2022 and December 2023.

The control group consisted of age- and sex-matched healthy children who attended the general pediatric outpatient clinic for routine health visits and had no history of neurodevelopmental, psychiatric, autoimmune, or chronic medical disorders. Controls were frequency-matched to the ASD group by age (± 6 months for children <5 years; ± 1 year for children ≥5 years) and sex.

All participants were recruited in the same city and healthcare setting to reduce geographic and sociodemographic variability between groups.

### Inclusion criteria

Children aged 2–9 years with a DSM-5 diagnosis of ASD.

### Exclusion criteria

Children with medical conditions known to affect cognitive or neurological function—such as diabetes mellitus, rickets, atrophic gastritis, cancer, bipolar disorder, psychotic disorders, epilepsy, other neurological or autoimmune diseases, or significant visual or hearing impairments—were excluded.

Children receiving immunomodulatory therapy or psychotropic medications initiated within the previous three months were also excluded to minimize potential confounding effects on laboratory parameters.

ASD diagnoses were established by a board-certified child psychiatrist through clinical evaluation based on DSM-5 criteria.

Autistic regression was defined as loss of previously acquired language and/or social communication skills lasting at least three months, with onset between 15 and 30 months, determined by parental report and further evaluated through structured clinical interview and review of available developmental records.

The study was approved by the Ethics Committee for Non-Interventional Clinical Researches of Çukurova University Faculty of Medicine (08.04.2022; No: 122). Written informed consent was obtained from parents of all participants.

### Scales and evaluation

After collecting demographic information and detailed medical histories, the following scales were applied:

- Ankara Developmental Screening Inventory (AGTE): Assesses developmental levels (0–6 years) in language-cognitive, fine motor, gross motor, and social/self-care domains. Scores are converted to T scores (mean = 50, SD = 10). Scores between 40–60 are considered age-appropriate (42).
- Autism Behavior Checklist (ABC): A 57-item screening tool with five subscales. Turkish adaptation has a cutoff score of 39 (43).
- Modified Checklist for Autism in Toddlers (M-CHAT): A 23-item, parent-reported screening tool. In the Turkish version, ≥2 high-risk or ≥3 total positive responses indicate high risk for ASD (44, 45).

The ABC total score was used as the primary measure of behavioral symptom severity. ABC was selected because it was available for all participants, has a validated Turkish version, and enabled standardized quantification of behavioral symptoms across the study sample; however, it was interpreted as a screening-based severity proxy rather than a gold-standard clinician-rated severity instrument. M-CHAT was used solely as a screening and descriptive tool and was not included in correlation or other inferential analyses evaluating symptom severity. AGTE was used to assess developmental functioning and to describe the cognitive-developmental profile of participants.

All assessments were performed by a single trained clinician blinded to laboratory results (clinical assessments were performed before laboratory results were available). The assessor was not blinded to participant group because ASD diagnosis and control status were clinically apparent.

### Laboratory measurements

In all participants, laboratory assessments included Anti-Streptolysin O (ASO), vitamin D, vitamin B12, thyroid-stimulating hormone (TSH), ferritin, iron, folate, and Anti-GAD antibody titers.

Blood samples were obtained between 08:00 and 10:00 AM after overnight fasting to minimize diurnal variation.

•Sample collection: For TSH, vitamin B12, ASO, ferritin, iron, and folate, 10 mL of venous blood was collected in plain tubes. For complete blood count and vitamin D, 2 mL samples were collected in EDTA tubes. All samples were centrifuged at 2000 × g for 15–20 minutes, and serum was analyzed immediately or stored at −80°C until assay.

•Assay methods:

- Iron: Ferrozine–deproteinization method
- Vitamin D: High-performance liquid chromatography (HPLC)
- Vitamin B12, TSH, and folate: Chemiluminescence immunoassay (CLIA)
- ASO: Immunonephelometric kinetic method

C-reactive protein (CRP) was measured initially but was not included in the final statistical analysis due to lack of significant between-group differences.

•Anti-GAD antibody analysis: Samples were collected in vacuum-sealed gel tubes (Becton Dickinson). After centrifugation, serum Anti-GAD levels were measured using a commercial ELISA kit (MEDIPAN, LOT: 22380204/1) according to the manufacturer’s instructions. Serum samples were analyzed undiluted. Calibrators, positive and negative controls, and patient samples were loaded into wells, followed by incubation with GAD65-biotin, streptavidin–peroxidase (SA-POD), and substrate solution. The reaction was stopped with 50 μL stop solution, and absorbance was measured at 450 nm. Concentrations <5 IU/mL were defined as negative, and ≥5 IU/mL as positive.

In addition to continuous analysis of Anti-GAD titers, Anti-GAD positivity rates were calculated and compared between groups. Both approaches were pre-specified to evaluate quantitative differences and potential clinical categorization. The intra- and inter-assay coefficients of variation reported by the manufacturer were <10%. All laboratory personnel were blinded to the clinical status and group allocation of the participants throughout sample processing and analysis.

### Statistical analysis

All statistical analyses were conducted using IBM SPSS Statistics version 26.0 (IBM Corp., Armonk, NY, USA). Categorical variables were expressed as frequencies and percentages, whereas continuous variables were summarized as means ± standard deviations for normally distributed data or medians with interquartile ranges for non-normally distributed data. Normality was assessed using the Shapiro–Wilk test.

Categorical variables were compared using the Chi-square test or Fisher’s exact test when expected cell counts were below 5. For continuous variables, independent-samples t-tests were applied if the assumptions of normality and homogeneity of variance were met; otherwise, the Mann–Whitney U test was used.

Correlations between continuous variables were examined using Pearson’s correlation coefficient for normally distributed data and Spearman’s rank correlation coefficient for non-normally distributed data. Analyses were limited to group comparisons and correlation testing in accordance with the exploratory design of the study.

A two-tailed p-value of <0.05 was considered statistically significant.

Given the exploratory nature of the study and the modest sample size, findings were interpreted cautiously. Post-hoc power calculations were not emphasized because of the recognized limitations of retrospective power estimation in observational studies.

## Results

A total of 90 participants were enrolled in the study, including 45 children diagnosed with ASD according to DSM-5 criteria and 45 healthy controls. The mean age was 48.3 months in the ASD group and 54.6 months in the control group. There were no statistically significant differences between groups in terms of age or sex distribution. In the ASD group, 37 children (82.2%) were male and 8 (17.8%) were female, whereas in the control group, 33 (73.3%) were male and 12 (26.7%) were female.

The demographic and clinical characteristics of the study sample are summarized in **Table 1**.

**Table 1:** Demographic and clinical characteristics of children with ASD and healthy controls.

Characteristic	ASD (n = 45)	Control (n = 45)	p-value
Age (months), mean ± SD	48.3 ± 20.1	54.6 ± 19.4	0.251
Male, n (%)	37 (82.2%)	33 (73.3%)	0.138
Female, n (%)	8 (17.8%)	12 (26.7%)	0.138
Autistic regression, n (%)	28 (62.2%)	Not applicable	—
No regression, n (%)	17 (37.8%)	Not applicable	—

Data are presented as mean ± standard deviation or n (%).

ASD, autism spectrum disorder.

p-values reflect comparisons between ASD and control groups.

Percentages are calculated within each study group.

The detailed laboratory findings for both groups are summarized in **Table 2**.

**Table 2:** Laboratory data of children with autism spectrum disorder and healthy controls.

Measurement	Total (n = 90) Mean ± SD	ASD (n = 45) Mean ± SD	Control (n = 45) Mean ± SD	p-value
Anti-GAD (IU/mL)	6.0 ± 0.9	6.3 ± 0.7	5.7 ± 1.0	0.003
ASO (IU/mL)	86.7 ± 114.5	102.2 ± 139.7	63.6 ± 53.9	0.399
Vitamin B12 (pg/mL)	321.2 ± 148.5	331.6 ± 153.4	306.5 ± 142.4	0.470
Vitamin D (ng/mL)	22.0 ± 10.0	20.2 ± 9.2	23.8 ± 10.6	0.096
Iron (µg/dL)	73.9 ± 35.0	69.9 ± 29.8	78.3 ± 40.0	0.293
Ferritin (ng/mL)	24.7 ± 21.1	25.3 ± 18.8	26.1 ± 23.5	0.862
TSH (µIU/mL)	2.2 ± 0.9	2.3 ± 1.0	2.1 ± 0.8	0.299

Data are presented as mean ± standard deviation (SD).

ASD, autism spectrum disorder; TSH, thyroid-stimulating hormone.

Among children with autism spectrum disorder, 62% had autistic regression. Compared with those without regression, children with regression had higher anti-glutamic acid decarboxylase titers and Autism Behavior Checklist total scores but lower iron and ferritin levels (all p < 0.05).

No statistically significant differences were observed in the other laboratory parameters.

Anti-GAD titers were positively correlated with ABC total scores (r = 0.724, p = 0.01). Given the sample size and the number of comparisons performed, this finding should be interpreted as exploratory rather than confirmatory.

The detailed results are presented in **Table 3**.

**Table 3:** Comparison of children with and without autistic regression in the ASD group.

Measurement	With regression (n = 28) Mean ± SD	Without regression (n = 17) Mean ± SD	p-value
Anti-GAD (IU/mL)	6.6 ± 0.69	5.9 ± 0.66	0.020*
ASO (IU/mL)	99.6 ± 142	107.3 ± 139.6	0.865
Vitamin B12 (pg/mL)	328.9 ± 156	337 ± 153.3	0.616
Vitamin D (ng/mL)	18.7 ± 9.2	23.4 ± 8.6	0.102
Iron (µg/dL)	58.9 ± 21.6	86.2 ± 34.9	0.03*
Ferritin (ng/mL)	22 ± 12.3	38.5 ± 24	0.04*
Folate (ng/mL)	13.1 ± 4.9	12.4 ± 3.4	0.630
TSH (µIU/mL)	2.3 ± 1.1	2.2 ± 0.8	0.670
ABC Total Score	68.6 ± 23.2	52.9 ± 20.5	0.024*
AGTE Total Score	91.3 ± 21.2	97.9 ± 22	0.408

Data are presented as mean ± standard deviation (SD). *p* < 0.05 was considered statistically significant. Abbreviations: TSH, thyroid-stimulating hormone; ABC, Autism Behavior Checklist; AGTE, Ankara Developmental Screening Inventory.

Laboratory assessments included Anti-GAD antibody titers, ASO, vitamin B12, serum iron, ferritin, and vitamin D. Anti-GAD antibody levels were significantly higher in children with ASD compared to healthy controls (p = 0.003). However, the absolute difference between groups was relatively small, and its clinical significance should be interpreted with caution.

In addition to continuous comparisons, Anti-GAD positivity (≥5 IU/mL) was evaluated; categorical positivity rates did not significantly differ between groups (p > 0.05).

## Discussion

In this study, we compared Anti-GAD antibody levels between children with ASD and healthy controls and examined their relationship with psychometric measures of ASD severity. We also analyzed laboratory and psychometric results in ASD subgroups with and without regression. Furthermore, we assessed the associations of iron, vitamin D, and vitamin B12—cofactors in key neurobiochemical pathways—with ASD.

According to our findings, Anti-GAD antibody levels were significantly higher in children with ASD than in controls (p = 0.003). However, the absolute difference between groups was modest, and its clinical relevance remains uncertain. In addition, although continuous Anti-GAD titers differed between groups, categorical Anti-GAD positivity rates did not significantly differ. This discrepancy suggests that the observed association may reflect a modest quantitative shift in antibody levels rather than a clinically meaningful seropositive subgroup. Previous studies have reported inconsistent results. Rout et al. (2012) found positivity in 20% of children with ADHD and 15% with ASD, while Kalra et al. (2015) reported that 98% of ASD cases were seronegative (46, 47). Other studies demonstrated reactivity of recombinant GAD65 antibodies with Purkinje cells, and postmortem analyses revealed reduced expression of both GAD65 and GAD67 in the cerebellum and parietal cortex of ASD patients (48–50).

The role of Anti-GAD antibodies in neurodevelopmental disorders remains uncertain. Although experimental data suggest that anti-GAD antibodies may influence GABAergic signaling, direct evidence demonstrating blood–brain barrier penetration or causal reduction of GABA levels in children with ASD is currently lacking. Therefore, our findings should be interpreted as statistical associations rather than mechanistic confirmation.

While prior experimental and animal studies have proposed potential links between GAD-related pathways and excitatory–inhibitory imbalance, the present case–control design does not allow conclusions regarding blood–brain barrier dynamics, neuronal excitotoxicity, or direct pathogenic mechanisms in ASD. The translational relevance of circulating Anti-GAD titers in children with ASD therefore remains to be clarified.

In addition, we explored the role of streptococcal infection by assessing ASO titers. Although children with ASD had higher mean ASO levels than controls, the difference was not statistically significant (p = 0.399). Literature on this association is scarce. Pozzi et al. reported elevated ASO titers in 18 of 28 ASD patients, and other studies suggest that age and timing of infection may influence ASO values (51–53). Further research, including microbial cultures and broader pathogen screening, is needed.

Regarding vitamin and mineral status, vitamin D levels were lower in ASD than in controls, but the difference did not reach significance (p = 0.096). Prior large-scale studies have reported decreased iron, vitamin D, and ferritin in ASD (54–57). In our study, mean iron and ferritin were slightly lower in ASD but not significantly different, while vitamin B12 and TSH levels were slightly higher. These findings highlight the metabolic variability observed in ASD rather than indicating a consistent immunometabolic pattern (58, 59).

When comparing ASD subgroups, children with regression (62%) showed significantly higher Anti-GAD titers and ABC scores but lower iron and ferritin levels (p < 0.05). The relatively high regression rate may reflect clinic-based sampling and the operational definition used.

These subgroup findings suggest a possible association between immunological markers and regression-related symptom burden; however, they should be interpreted cautiously and cannot establish causality (60–62).

A significant positive correlation was observed between Anti-GAD titers and ABC total score (r = 0.724, p = 0.01). Given the relatively small sample size and the number of statistical comparisons performed, this correlation should be interpreted as exploratory and hypothesis-generating rather than confirmatory.

Strengths of our study include the use of a control group, strict exclusion criteria, assessment by a single clinician, comprehensive biochemical analyses, and a relatively larger sample size compared to previous reports (63–66). Limitations include the lack of a community-based control group, reliance on parent-reported scales for symptom severity, and measurement of vitamin D at different times of the year, which may have influenced variability.

## Conclusions and recommendations

The findings of this study indicate that elevated Anti-GAD antibody levels are statistically associated with ASD and with the presence of autistic regression. However, given the modest absolute differences observed and the cross-sectional design of the study, these findings should be interpreted as exploratory associations rather than evidence of etiopathogenic involvement.

At present, the available data do not support the clinical use of Anti-GAD antibodies as diagnostic or prognostic biomarkers in clinical practice. Further large-scale, longitudinal, and multicenter studies are required to determine whether immunological parameters contribute meaningfully to ASD heterogeneity.

Given the high rate of regression observed in our study, we recommend that regression features be carefully assessed in all children with ASD. Future research should aim to integrate immunological, biochemical, and behavioral data within well-defined phenotypic subgroups to better understand potential biological variability.

Future research with larger, community-based, multicenter, and longitudinal samples is needed to clarify the relationship between immunological and biochemical factors in ASD. Such studies should integrate clinical data, psychometric scales, and laboratory parameters in both ASD and control populations.

Although immune-related mechanisms have been proposed in subsets of neuropsychiatric conditions (67–70), the present study does not specifically address acute-onset presentations, and extrapolation to frameworks such as Pediatric Acute-onset Neuropsychiatric Syndrome (PANS) should therefore be made with caution.

To enhance the reliability of findings, parent-reported measures should be supplemented with objective data sources such as teacher reports, video recordings, and observational assessments in naturalistic settings. Finally, evaluating biochemical parameters together with related enzymes and cofactors could provide a more comprehensive understanding of underlying neurobiological mechanisms (71–73).

## Limitations

This study provides preliminary evidence regarding immune-related associations in ASD; however, several important limitations should be acknowledged. First, the control group was hospital-based rather than community-based, which may restrict the generalizability of our findings. Selection bias cannot therefore be fully excluded.

Second, the severity of autism symptoms was assessed primarily through parent-reported questionnaires, which may introduce subjectivity, recall bias, and reporting bias. Objective clinician-rated or observational measures were not systematically incorporated.

Third, vitamin D levels were measured at different times of the year, making the results potentially susceptible to seasonal variation as well as dietary and lifestyle influences.

Fourth, the cross-sectional design precludes any inference regarding temporal relationships or causality between Anti-GAD titers and ASD-related features.

Finally, the relatively small sample size may have limited statistical power and increased the risk of both type I and type II errors. In addition, multiple statistical comparisons were performed, which further increases the possibility of false-positive findings.

### Strengths

The inclusion of an age- and sex-matched control group represents a key strength of this study. Additionally, the use of strict exclusion criteria minimized the potential influence of comorbid conditions on both laboratory and clinical outcomes. All participants were evaluated by a single clinician, which ensured diagnostic consistency for ASD and reliable assessment of autistic regression. Blinding of laboratory personnel to clinical status further reduced measurement bias. Another strength lies in the comprehensive evaluation of biochemical parameters, allowing integrated assessment of immunological and metabolic variables within the same cohort. Although the sample size was modest, it is comparable to or larger than several prior exploratory studies in this field. Therefore, the findings may be considered reasonably robust within the context of an exploratory case–control design.

## Data Availability

The raw data supporting the conclusions of this article will be made available by the authors, without undue reservation.

## References

[1] American Psychiatric Association . Diagnostic and statistical manual of mental disorders. Washington DC: American Psychiatric Publishing (2013).

[2] Kanner L . Autistic disturbances of affective contact. Nerv Child. (1943) 2:217–50. 4880460

[3] Maenner MJ Shaw KA Bakian AV Bilder DA Durkin MS Esler A . Prevalence and characteristics of autism spectrum disorder among children aged 8 years—Autism and Developmental Disabilities Monitoring Network, 11 sites, United States, 2018. MMWR Surveill Summ. (2021) 70:1–16. doi: 10.15585/mmwr.ss7011a1. PMID: 34855725 PMC8639024

[4] Fombonne E . Epidemiology of pervasive developmental disorders. Pediatr Res. (2009) 65:591–8. doi: 10.1203/PDR.0b013e31819e7203. PMID: 19218885

[5] Szatmari P Jones MB . IQ and the genetics of autism. J Child Psychol Psychiatry. (1991) 32:897–908. doi: 10.1111/j.1469-7610.1991.tb01917.x. PMID: 1744193

[6] Treffert DA . Epidemiology of infantile autism. Arch Gen Psychiatry. (1970) 22:431–8. doi: 10.1001/archpsyc.1970.01740290047006. PMID: 5436867

[7] Volkmar FR Rutter M . Childhood disintegrative disorder: results of the DSM-IV autism field trial. J Am Acad Child Adolesc Psychiatry. (1995) 34:1092–5. doi: 10.1097/00004583-199508000-00020. PMID: 7665448

[8] Şener EF Özkul Y . Sağlık bilim derg, vol. 22. (2013). DergiPark, Ankara, Türkiye. p. 86–92. Available online at: https://dergipark.org.tr/tr/pub/eujhs/issue/44556/552741 (Accessed October 10, 2025).

[9] Kubota T Miyake K Hariya N Mochizuki K . Epigenetics as a basis for diagnosis of neurodevelopmental disorders: challenges and opportunities. Expert Rev Mol Diagn. (2014) 14:685–97. doi: 10.1586/14737159.2014.925805. PMID: 24902600

[10] Mandy W Lai MC . Annual research review: the role of the environment in the developmental psychopathology of autism spectrum condition. J Child Psychol Psychiatry. (2016) 57:271–92. doi: 10.1111/jcpp.12501. PMID: 26782158

[11] Idring S Magnusson C Lundberg M Ek M Rai D Svensson AC . Parental age and the risk of autism spectrum disorders: findings from a Swedish population-based cohort. Int J Epidemiol. (2014) 43:107–15. doi: 10.1093/ije/dyt262. PMID: 24408971

[12] Lyall K Croen L Daniels J . The changing epidemiology of autism spectrum disorders. Annu Rev Public Health. (2017) 38:81–102. doi: 10.1146/annurev-publhealth-031816-044318. PMID: 28068486 PMC6566093

[13] Croen LA Grether JK Yoshida CK Odouli R Hendrick V . Antidepressant use during pregnancy and childhood autism spectrum disorders. Arch Gen Psychiatry. (2011) 68:1104–12. doi: 10.1001/archgenpsychiatry.2011.73. PMID: 21727247

[14] Ozonoff S Heung K Byrd R Hansen R Hertz-Picciotto I . The onset of autism: patterns of symptom emergence in the first years of life. Autism Res. (2008) 1:320–8. doi: 10.1002/aur.53. PMID: 19360687 PMC2857525

[15] Fombonne E . Prevalence of childhood disintegrative disorder. Autism. (2002) 6:149–57. doi: 10.1177/1362361302006002002. PMID: 12083281

[16] Barger BD Campbell JM McDonough JD . Prevalence and onset of regression within autism spectrum disorders: a meta-analytic review. J Autism Dev Disord. (2013) 43:817–28. doi: 10.1007/s10803-012-1621-x. PMID: 22855372

[17] Gliga T Jones EJH Bedford R Charman T Johnson MH . From early markers to neurodevelopmental mechanisms of autism. Dev Rev. (2014) 34:189–207. doi: 10.1016/j.dr.2014.05.003. PMID: 25187673 PMC4119302

[18] Fournier KA Hass CJ Naik SK Lodha N Cauraugh JH . Motor coordination in autism spectrum disorders: a synthesis and meta-analysis. J Autism Dev Disord. (2010) 40:1227–40. doi: 10.1007/s10803-010-0981-3. PMID: 20195737

[19] Ackermann H Wildgruber D Daum I Grodd W . Does the cerebellum contribute to cognitive aspects of speech production? Neurosci Lett. (1998) 247:187–90. doi: 10.1016/S0304-3940(98)00328-0. PMID: 9655624

[20] Shriberg LD Paul R Black LM Van Santen JP . The hypothesis of apraxia of speech in children with autism spectrum disorder. J Autism Dev Disord. (2011) 41:405–26. doi: 10.1007/s10803-010-1117-5. PMID: 20972615 PMC3033475

[21] Schmahmann JD . The cerebellum and cognition. Neurosci Lett. (2019) 688:62–75. doi: 10.1016/j.neulet.2018.07.005. PMID: 29997061

[22] Courchesne E . Brainstem, cerebellar and limbic neuroanatomical abnormalities in autism. Curr Opin Neurobiol. (1997) 7:269–78. doi: 10.1016/S0959-4388(97)80016-5. PMID: 9142760

[23] Whitney ER Kemper TL Bauman ML Rosene DL Blatt GJ . Cerebellar Purkinje cells are reduced in a subpopulation of autistic brains. Cerebellum. (2008) 7:406–16. doi: 10.1007/s12311-008-0043-y. PMID: 18587625

[24] Cohly HHP Panja A . Immunological findings in autism. Int Rev Neurobiol. (2005) 71:317–41. doi: 10.1016/S0074-7742(05)71013-8. PMID: 16512356

[25] Pardo CA Eberhart CG . The neurobiology of autism. Brain Pathol. (2007) 17:434–47. doi: 10.1111/j.1750-3639.2007.00102.x. PMID: 17919129 PMC8095519

[26] Schmahmann JD Rosene DL Pandya DN . Motor projections to the basis pontis in rhesus monkey. J Comp Neurol. (2004) 478:248–68. doi: 10.1002/cne.20286. PMID: 15368534

[27] Nowinski CV Minshew NJ Luna B Takarae Y Sweeney JA . Oculomotor studies of cerebellar function in autism. Psychiatry Res. (2005) 137:11–9. doi: 10.1016/j.psychres.2005.07.005. PMID: 16214219

[28] Solimena M De Camilli P . Autoimmunity to glutamic acid decarboxylase (GAD) in stiff-man syndrome and insulin-dependent diabetes mellitus. Trends Neurosci. (1991) 14:452–7. doi: 10.1016/0166-2236(91)90044-U. PMID: 1722364

[29] Kaufman DL Houser R Tobin AJ . Two forms of the γ-aminobutyric acid synthetic enzyme glutamate decarboxylase have distinct intraneuronal distributions. Science. (1990) 248:720–3. doi: 10.1111/j.1471-4159.1991.tb08211.x. PMID: 1988566 PMC8194030

[30] Pinal CS Tobin AJ . Uniqueness and redundancy in GABA production. Perspect Dev Neurobiol. (1998) 5:109–18. doi: 10.1111/j.1750-3639.2007.00102.x, PMID: 9777629

[31] Schmahmann JD Rosene DL Pandya DN . Motor projections to the basis pontis in rhesus monkey. J Comp Neurol. (2004) 478:248–268. doi: 10.1002/cne.20286, PMID: 15368534

[32] Nowinski CV Minshew NJ Luna B Takarae Y Sweeney JA . Oculomotor studies of cerebellar function in autism. Psychiatry Res. (2005) 137:11–19. doi: 10.1016/j.psychres.2005.07.005, PMID: 16214219

[33] Fouka P Alexopoulos H Akrivou S Trohatou O Politis PK Dalakas MC . GAD65 epitope mapping and search for novel autoantibodies in GAD-associated neurological disorders. J Neuroimmunol. (2015) 281:73–7. doi: 10.1016/j.jneuroim.2015.03.009. PMID: 25867471

[34] Watanabe M Maemura K Kanbara K Tamayama T Hayasaki H . GABA and GABA receptors in the central nervous system and other organs. Int Rev Cytol. (2002) 213:1–47. doi: 10.1016/S0074-7696(02)13011-7. PMID: 11837891

[35] Kern JK . Purkinje cell vulnerability and autism: a possible etiological connection. Brain Dev. (2003) 25:377–82. doi: 10.1016/S0387-7604(03)00056-1. PMID: 12907269

[36] Pierce K Courchesne E . Evidence for a cerebellar role in reduced exploration and stereotyped behavior in autism. Biol Psychiatry. (2001) 49:655–64. doi: 10.1016/S0006-3223(00)01008-8. PMID: 11313033

[37] Jones RSG . Epileptiform events induced by GABA antagonists in entorhinal cortical cells *in vitro* are partly mediated by N-methyl-D-aspartate receptors. Brain Res. (1988) 457:113–21. doi: 10.1016/0006-8993(88)90062-5. PMID: 2901894

[38] Erol N Sezgin N Savaşır I . Gelişim tarama envanteri ile ilgili geçerlilik çalışmaları. Türk Psikol Derg. (1993) 8:16–22. doi: 10.1016/j.jneuroim.2015.03.009, PMID: 25867471

[39] Irmak YT Sütcü TS Aydın A Sorias O . Otizm Davranış Kontrol Listesinin geçerlilik ve güvenilirliğinin incelenmesi. Çocuk ve Gen Ruh Sağlığı Derg. (2007) 14:13–23. doi: 10.1016/S0074-7696(02)13011-7, PMID: 11837891

[40] Robins DL Fein D Barton ML Green JA . The Modified Checklist for Autism in Toddlers: an initial study investigating early detection. J Autism Dev Disord. (2001) 31:131–44. doi: 10.1023/A:1010738829569. PMID: 11450812

[41] Kara B Mukaddes NM Altinkaya I Güntepe D Gökçay G Özmen M . Using the Modified Checklist for Autism in Toddlers in a well-child clinic in Turkey. Autism. (2014) 18:331–8. doi: 10.1177/1362361312467864. PMID: 23175752

[42] Rout UK Mungan NK Dhossche DM . Presence of GAD65 autoantibodies in the serum of children with autism or ADHD. Eur Child Adolesc Psychiatry. (2012) 21:141–7. doi: 10.1007/s00787-012-0245-1. PMID: 22323074

[43] Erol N Sezgin N Savaşır I . Ankara gelişim tarama envanteri ile ilgili geçerlik çalışmaları. Türk Psikol Derg. (1993) 8:16–22.

[44] Vianello M Keir G Giometto B Betterle C Tavolato B Thompson EJ . Antigenic differences between neurological and diabetic patients with anti-glutamic acid decarboxylase antibodies. Eur J Neurol. (2005) 12:294–9. doi: 10.1111/j.1468-1331.2004.00933.x. PMID: 15804247

[45] Fatemi SH Halt AR Stary JM Kanodia R Schulz SC Realmuto GR . Glutamic acid decarboxylase 65 and 67 kDa proteins are reduced in autistic parietal and cerebellar cortices. Biol Psychiatry. (2002) 52:805–10. doi: 10.1016/S0006-3223(02)01430-0. PMID: 12372652

[46] Rout UK Dhossche DM . A pathogenetic model of autism involving Purkinje cell loss through anti-GAD antibodies. Med Hypotheses. (2008) 71:218–21. doi: 10.1016/j.mehy.2007.11.012. PMID: 18514431

[47] Aslan C Konuşkan B Şener B Ünal F . Comparison of serum anti-neuronal antibody levels in patients having autism spectrum disorder with and without regression. Turk J Pediatr. (2021) 63:780–9. doi: 10.24953/turkjped.2021.05.006. PMID: 34738360

[48] Malter MP Helmstaedter C Urbach H Vincent A Bien CG . Antibodies to glutamic acid decarboxylase define a form of limbic encephalitis. Ann Neurol. (2010) 67:470–8. doi: 10.1002/ana.21917. PMID: 20437582

[49] Jones KL Pride MC Edmiston E . Autism-specific maternal autoantibodies produce behavioral abnormalities in an endogenous antigen-driven mouse model of autism. Mol Psychiatry. (2020) 25:2994–3009. doi: 10.1038/s41380-018-0126-1. PMID: 29955164 PMC6310680

[50] Manto MU Laute MA Aguera M Rogemond V Pandolfo M Honnorat J . Effects of anti-glutamic acid decarboxylase antibodies associated with neurological diseases. Ann Neurol. (2007) 61:544–51. doi: 10.1002/ana.21123. PMID: 17600364

[51] Guo M Li L Zhang Q . Vitamin and mineral status of children with autism spectrum disorder in Hainan Province of China: associations with symptoms. Nutr Neurosci. (2020) 23:803–10. doi: 10.1080/1028415X.2018.1558762. PMID: 30570388

[52] Liu X Liu J Xiong X Yang T Hou N Liang X . Correlation between nutrition and symptoms: nutritional survey of children with autism spectrum disorder in Chongqing, China. Nutrients. (2016) 8:294. doi: 10.3390/nu8050294. PMID: 27187463 PMC4882707

[53] Ali A Waly M Al-Farsi Y Essa M Al-Sharbati M Deth R . Hyperhomocysteinemia among Omani autistic children: a case-control study. Acta Biochim Pol. (2011) 58:547–51. doi: 10.18388/abp.2011_2223 22187679

[54] Meltzer A Van De Water J . The role of the immune system in autism spectrum disorder. Neuropsychopharmacology. (2017) 42:284–98. doi: 10.1038/npp.2016.158. PMID: 27534269 PMC5143489

[55] Schmidt RJ Hansen RL Hartiala J Allayee H Schmidt LC Tancredi DJ . Prenatal vitamins, one-carbon metabolism gene variants, and risk for autism. Epidemiology. (2011) 22:476–85. doi: 10.1097/EDE.0b013e31821d0e30. PMID: 21610500 PMC3116691

[56] DeVilbiss EA Gardner RM Newschaffer CJ Lee BK . Maternal folate status as a risk factor for autism spectrum disorders: a review of existing evidence. Br J Nutr. (2015) 114:663–72. doi: 10.1017/S0007114515002470. PMID: 26243379

[57] Frye RE Nankova B Bhattacharyya S Rose S Bennuri SC MacFabe DF . Modulation of immunometabolism and neurotransmission in autism spectrum disorders by gut microbiota. Front Neurosci. (2019) 13:1049. doi: 10.3389/fnins.2019.01049. PMID: 31636531 PMC6787308

[58] Bjørklund G Meguid NA El-Bana MA . Oxidative stress in autism spectrum disorder. Mol Neurobiol. (2020) 57:2312–22. doi: 10.1007/s12035-019-01742-2. PMID: 32026227

[59] Frustaci A Neri M Cesario A Adams JB Domenici E Dalla Bernardina B . Oxidative stress-related biomarkers in autism: systematic review and meta-analyses. Free Radic Biol Med. (2012) 52:2128–41. doi: 10.1016/j.freeradbiomed.2012.03.011. PMID: 22542447

[60] Vorstman JAS Parr JR Moreno-De-Luca D Anney RJL Nurnberger JI Hallmayer JF . Autism genetics: opportunities and challenges for clinical translation. Nat Rev Genet. (2017) 18:362–76. doi: 10.1038/nrg.2017.4. PMID: 28260791

[61] Kočovská E Fernell E Billstedt E Minnis H Gillberg C . Vitamin D and autism: clinical review. Res Dev Disabil. (2012) 33:1541–50. doi: 10.1016/j.ridd.2012.02.015. PMID: 22522213

[62] Miodovnik A Engel SM Zhu C . Endocrine disruptors and childhood social impairment. Neurotoxicology. (2011) 32:261–7. doi: 10.1016/j.neuro.2010.12.009. PMID: 21182865 PMC3057338

[63] Gardener H Spiegelman D Buka SL . Prenatal risk factors for autism: comprehensive meta-analysis. Br J Psychiatry. (2009) 195:7–14. doi: 10.1192/bjp.bp.108.051672. PMID: 19567888 PMC3712619

[64] Dufour-Rainfray D Vourc’h P Tourlet S Guilloteau D Chalon S Andres CR . Fetal exposure to teratogens: evidence of genes involved in autism. Neurosci Biobehav Rev. (2011) 35:1254–65. doi: 10.1016/j.neubiorev.2010.12.013. PMID: 21195109

[65] Manto M Dalmau J Didelot A Rogemond V Honnorat J . *In vivo* effects of antibodies from patients with anti-NMDA receptor encephalitis: further evidence of synaptic glutamatergic dysfunction. Orphanet J Rare Dis. (2010) 5:31. doi: 10.1186/1750-1172-5-31. PMID: 21110857 PMC3002330

[66] Antonini G Nemni R Giubilei F . Autoantibodies to glutamic acid decarboxylase in downbeat nystagmus. J Neurol Neurosurg Psychiatry. (2003) 74:998–9. doi: 10.1136/jnnp.74.7.998. PMID: 12810806 PMC1738554

[67] Blatt GJ . GABAergic cerebellar system in autism: a neuropathological and developmental perspective. Int Rev Neurobiol. (2005) 71:167–78. doi: 10.1016/S0074-7742(05)71007-2. PMID: 16512350

[68] Swedo SE . From research subgroup to clinical syndrome: modifying the PANDAS criteria to describe PANS (pediatric acute-onset neuropsychiatric syndrome). Pediatr Ther. (2012) 2:1000113. doi: 10.4172/2161-0665.1000113. PMID: 39887974

[69] Pozzi M Pisano S Bertella S Lombardi P Pellegrino P Molteni M . On the possible relationship between anti-streptolysin-O titer and neuropsychiatric disorders other than PANS. J Child Adolesc Psychopharmacol. (2015) 25:452–6. doi: 10.1089/cap.2014.0089. PMID: 26091201

[70] Murphy TK Patel PD McGuire JF . Characterization of the pediatric acute-onset neuropsychiatric syndrome phenotype. J Child Adolesc Psychopharmacol. (2015) 25:14–25. doi: 10.1089/cap.2014.0062. PMID: 25314221 PMC4340632

[71] Kaplan EL Rothermel CD Johnson DR . Antistreptolysin O and anti-deoxyribonuclease B titers: normal values for children ages 2 to 12 in the United States. Pediatrics. (1998) 101:86–8. doi: 10.1542/peds.101.1.86. PMID: 9417157

[72] Bener A Khattab AO Bhugra D Hoffmann GF . Iron and vitamin D levels among autism spectrum disorder children. Ann Afr Med. (2017) 16:186–91. doi: 10.4103/aam.aam_17_17. PMID: 29063903 PMC5676409

[73] Gunes S Ekinci O Celik T . Iron deficiency parameters in autism spectrum disorder: clinical correlates and associated factors. Ital J Pediatr. (2017) 43:86. doi: 10.1186/s13052-017-0407-3. PMID: 28934988 PMC5609017

